# Protective effect of HDL on NADPH oxidase-derived super oxide anion mediates hypoxia-induced cardiomyocyte apoptosis

**DOI:** 10.1371/journal.pone.0179492

**Published:** 2017-06-15

**Authors:** Su-Ying Wen, Shanmugam Tamilselvi, Chia-Yao Shen, Cecilia Hsuan Day, Li-Chin Chun, Li-Yi Cheng, Hsiu-Chung Ou, Ray-Jade Chen, Vijaya Padma Viswanadha, Wei-Wen Kuo, Chih-Yang Huang

**Affiliations:** 1 Department of Dermatology, Taipei City Hospital, Renai Branch, Taipei, Taiwan; 2 Center for General Education, Mackay Junior College of Medicine, Nursing, and Management, Taipei, Taiwan; 3 Graduate Institute of Basic Medical Science, China Medical University, Taichung, Taiwan; 4 Department of Nursing, MeiHo University, Pingtung, Taiwan; 5 Department of Hospital and Health Care Administration, Chia Nan University of Pharmacy & Science, Tainan County, Taiwan; 6 Department of Biological Science and Technology, Asia University, Taichung, Taiwan; 7 Department of Surgery, School of Medicine, College of Medicine, Taipei Medical University, Taipei, Taiwan; 8 Department of Biotechnology, Bharathiar University, Coimbatore, India; 9 Department of Biological Science and Technology, China Medical University, Taichung, Taiwan; 10 Graduate Institute of Chinese Medical Science, China Medical University, Taichung, Taiwan; National Institutes of Health, UNITED STATES

## Abstract

Cardiovascular diseases are the leading cause of death of death in Taiwan. Atherosclerosis can lead to serious problems, including heart attack, stroke, or even death. Coronary heart disease (CHD) occurs when plaque builds up in the coronary arteries to cause the ischemic heart disease which will enhance myocardial remodeling and also induce myocardial hypoxia. High density lipoprotein (HDL) has been proposed to have cardio-protective effects. Under hypoxic conditions (1%O_2_ for 24hr), in H9c2 cells, reactive oxygen species (ROS) is induced which leads to cardiomyocyte apoptosis and cardiac dysfunction. Therefore, the present study described the protective effect of HDL on hypoxia-induced cardiomyocyte damage. We investigated the NADPH oxidase-produced ROS-related signaling pathways and apoptosis in cardiomyocytes under hypoxia conditions. Results showed that the ROS mediated cardiac damage might occur via AT1 and PKC activation. Furthermore, hypoxia downregulated the survival protein (p-AKT^ser473^) and anti-apoptotic protein (BCL_2_), whereas pro-apoptotic protein, Bax and caspase 3 were upregulated. These detrimental effects by ROS and apoptosis were prevented by HDL pretreatment. Our findings revealed the underlying molecular mechanism by which HDL suppresses the hypoxia-induced cardiomyocyte dysfunction. Further, we elucidated the role of HDL on preventing hypoxia induced cardiomyocyte apoptosis is mediated through the inhibition of NADPH oxidase-derived ROS.

## Introduction

Atherosclerosis is one of the top ten reasons of death in Taiwan caused due to the accumulation of fatty substances, cholesterol, cellular waste products, calcium and fibrin on the inner coating of artery[1]. Arteries are the blood vessels which deliver blood from the heart throughout the body. Formation of plaques in the arteries could cause chest pain on exertion, and also reduces the supply of oxygen to all the parts of body leading to hypoxic condition [2].

Under normoxic condition, the working heart produces an abundant supply of ATP (>95%), mainly from fat oxidation. Under anaerobic conditions, mammalian heart cells cannot generate enough energy to perform essential cellular functions; therefore, a continuous supply of oxygen is essential to retain the function and viability of heart [3]. Hypoxic/ischemic injury (I/R) is one of the major stresses of heart, leading to detrimental effects through the activation of fetal cardiac genes and specific signal transduction pathways [4]. Gene expression is altered by several mechanisms based on the availability of oxygen in the heart which includes the regulation of gene transcription by hypoxia-inducible factor 1α (HIF-1α) [5, 6]. HIF-1α mediates the transcription of many important genes, which can control vital cellular processes like vascular remodeling, metabolism, apoptosis, control of ROS, vasomotor reactivity, and inflammation [7–9].

Oxidative stress is caused by reactive oxygen species (ROS) which produces oxygen free radicals, that contain unpaired electrons and are highly reactive and short-lived [10]. Under normoxic condition, ROS can be formed by different mechanisms which includes the production through oxidative phosphorylation in the mitochondria as a byproduct of normal cellular metabolism in heart [11, 12].

In cardiomyocytes, hypoxia has reported to induce various harmful effects which include cell proliferation, cell hypertrophy and cell death. As a response to hypoxia, cardiomyocytes express a number of genes, that induced by a variety of signaling cascades [13–16].

High density lipoprotein (HDL) is a circulating-complex of lipids, bioactive particles, containing multiple acute phase response proteins, protease inhibitors, and complement regulatory proteins. Many reports have documented that HDL-cholesterol levels are inversely associated with the risk of cardiovascular diseases [17, 18]. The effect of HDL could be influenced by abundance of several bioactive proteins and lipids, and could exert anti-inflammatory, anti-oxidative, anti-coagulative and other atheroprotective functions [19].

Therefore, the aim of this study was to explore the underlying protective mechanisms of HDL against hypoxia-induced oxidative stress in cardiomyocytes. We investigated the ROS mediated activation and subsequent inflammatory and apoptotic signaling pathways.

## Materials and methods

### Cell culture

H9c2 cell lines were obtained from American Type Culture Collection (ATCC), cultured in Dulbecco’s modified essential medium (DMEM) supplemented with 10% Cosmic Calf serum (CCS), 2mM glutamine, 100units/ml penicillin, 100μg/ml streptomycin, and 1mM pyruvate in humidified air (5% CO_2_) at 37°C. During the treatment, cells were pretreated with HDL (25, 50 and 100μg/ ml) for 2 hours and then incubated in hypoxic chamber (1% O_2_ for 24 hours). The specificity of the inhibit ROS and mitochondria complex I inhibitor by adding N-acetly cysteine (NAC) (500μM).

### Reactive oxygen species and mitochondrial superoxide production

Intracellular generation of ROS was monitored by flow cytometry using peroxide-sensitive fluorescent probe 2′, 7′-dichlorofluorescein diacetate (DCFH-DA used as a Molecular Probes). The fluorescent dichlorofluorescein (DCF) formed by oxidation of DCFH was quantified by flow cytometry. Furthermore, mitochondrial superoxide (O2radical dot−) was evaluated by MitoSOX Red mitochondrial superoxide indicator (Invitrogen, Eugene, OR). Cells were harvested after treatment. Cells were resuspended with MitoSOX (2 μM) and incubated at 37°C for 10–15 min. Samples were analyzed by a BD FACSCantoM II flow cytometer (Becton—Dickinson, Franklin Lakes, NJ) to detect the mean mitochondrial ROS production.

### Immunoblotting

H9c2 cells were washed once with PBS and harvested, then cell suspension was spun down, and lysed in RIPA buffer (HEPES 20mM, MgCl2 1.5mM, EDTA 2mM, EGTA 5mM, dithiothreitol 0.1mM, phenylmethylsulfonyl fluoride 0.1mM, pH 7.5) for 4hr at 4°C. Total cell lysate was spun down 12,000 rpm for 20 min at 4°C and the supernatant was collected in new eppendorf tube. Total protein was estimated by Bradford’s method. Proteins (30 μg) were separated by electrophoresis on SDS-polyacrylamide gel. After the protein had been transferred to polyvinylidene difluoride (PVDF) membrane, the blots were incubated with blocking buffer (1X PBS and 5% nonfat dry milk) for 1 hour at room temperature and then probed with primary antibodies (1:1000 dilutions) overnight at 4°C, followed by incubation with horseradish peroxidase-conjugated secondary antibody (1:5000) for 1 hour. To control equal loading of total protein in all lanes, blots were stained with mouse anti-β-actin antibody at a 1:50000 dilutions. Antibodies AT 1 (sc-1173), P47 (sc-14015), p-PKCδ (sc-11776), Bcl 2 (sc-7382), Bax (sc-526), p-P38 (sc-7973), p-ERK (sc-7383), p-JNK (sc-6254), caspase 3 (sc-7148) and β-actin (sc-47778) and their respective secondary antibodies were purchased from Santa Cruz Biotechnology, CA. Antibodies gp41 (ab 31092) and Rac 1 (ab-33186) were purchased from ab cam, UK and pAkt (cs# 92755) was purchased from cell signaling Technology, Inc, USA. The bound immunoproteins were detected by an ECL kit.

### DAPI staining and TUNEL assay

After treatment H9c2 cells were grown on 6 mm plates. Then the cells were washed with PBS and were fixed with 4% paraformaldehyde solution for 30 min at room temperature. After a wash with PBS, cells were treated with permeation solution (0.1% Triton X-100 in 0.1% sodium citrate) for 2 min at 4°C. Following wash with PBS, samples were first incubated with TUNEL reagent containing terminal deoxynucleotidyl transferase and fluorescent isothiocyanate-dUTP. The cells were also stained with 1μg/ml DAPI for 30 min to detect cell nucleus by UV light microscopic observations (blue). Samples were analyzed in a drop of PBS under a fluorescence and UV light microscope, respectively. Apoptotic cells were calculated by fluorescence microscope.

### Neonatal cardiomyocyte culture

Neonatal cardiomyocytes were isolated and cultured using the commercial Neonatal Cardiomyocyte Isolation System Kit (Cellutron Life Technologies, Baltimore, MD, USA) according to manufacturer’s directions. Briefly, hearts were quickly removed from one- to two-days-old Sprague-Dawley decapitated rats, the ventricles were pooled, and the ventricular cells were dispersed by digestion solution at 37°C. Ventricular cardiomyocytes were isolated and cultured in DMEM containing 10% fetal bovine serum, 100μg/ml penicillin, 100μg/ml streptomycin, and 2 mM glutamine. Cells were incubated in serum-free essential medium overnight before treatment with indicated agents. All experimental procedures were performed according to the NIH guide for the care and use of laboratory animals. All protocols were approved by the Institutional Animal Care and Use Committee of China Medical University, Taichung, Taiwan.

### Statistical analysis

Statistical differences were assessed by one way-ANOVA or t-test. P < 0.05 was considered statistically significant. Data were expressed as the mean ± SEM.

## Result

### Sustained incubation of H9c2 cells under hypoxia conditions increases reactive oxygen species (ROS) production and decreases the expression of antioxidant enzyme

**Fig 1:**
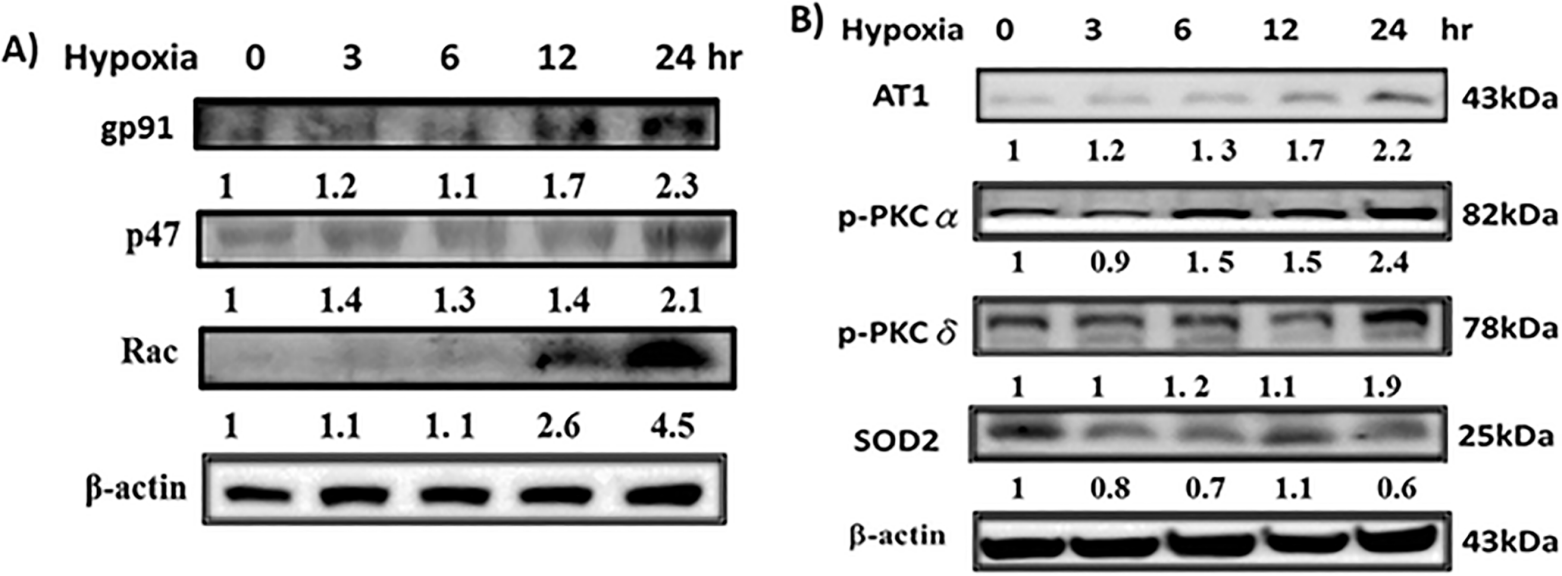
Hypoxia-increased oxidative stress in H9c2 cells. (A) The level of NADPH oxidase (Nox2-gp91 ^phox^, p47^phox^, and Rac1) protein expressions. (B) The level of AT1 receptor, p-PKCα and p-PKCδ, and antioxidant enzyme SOD2 protein expressions. ‘Image J’ software was used to calculate the expression level of protein.

Cardiovascular injury, one of the most common complications of hypoxia, is linked to the elevated ROS levels, which subsequently induce cell apoptosis. Therefore, we examined the cellular ROS levels in cardiomyoblast H9c2 cells incubated in sustained hypoxia conditions. The effects of hypoxia on the expression of gp91phox, p47phox and Rac-1 were observed by western blotting in H9c2 cells. We found that the protein levels of NADPH oxidases were increased in H9c2 cells exposed to hypoxia for 0-24h (Fig 1A).

As shown in Fig 1B, after treatment with hypoxia for different time periods, the protein expression levels of AT1, p-PKC-α and p-PKC -δ were increased in H9c2 cells. Intracellular ROS level has been regulated by the balance between ROS generation and the activity of antioxidant enzymes such as catalase or SOD. Thus, the involved ROS might inactivate anti-oxidative enzymes that additionally increase the imbalance in favor of oxidative stress. Therefore, we investigated the expression of its isoforms in H9c2 cells in response to hypoxia. Our results showed that the antioxidant enzymes SOD2 decreased in H9c2 cells treated with hypoxia (Fig 1B).

### Hypoxia-induced apoptosis in cardiomyocyte

**Fig 2:**
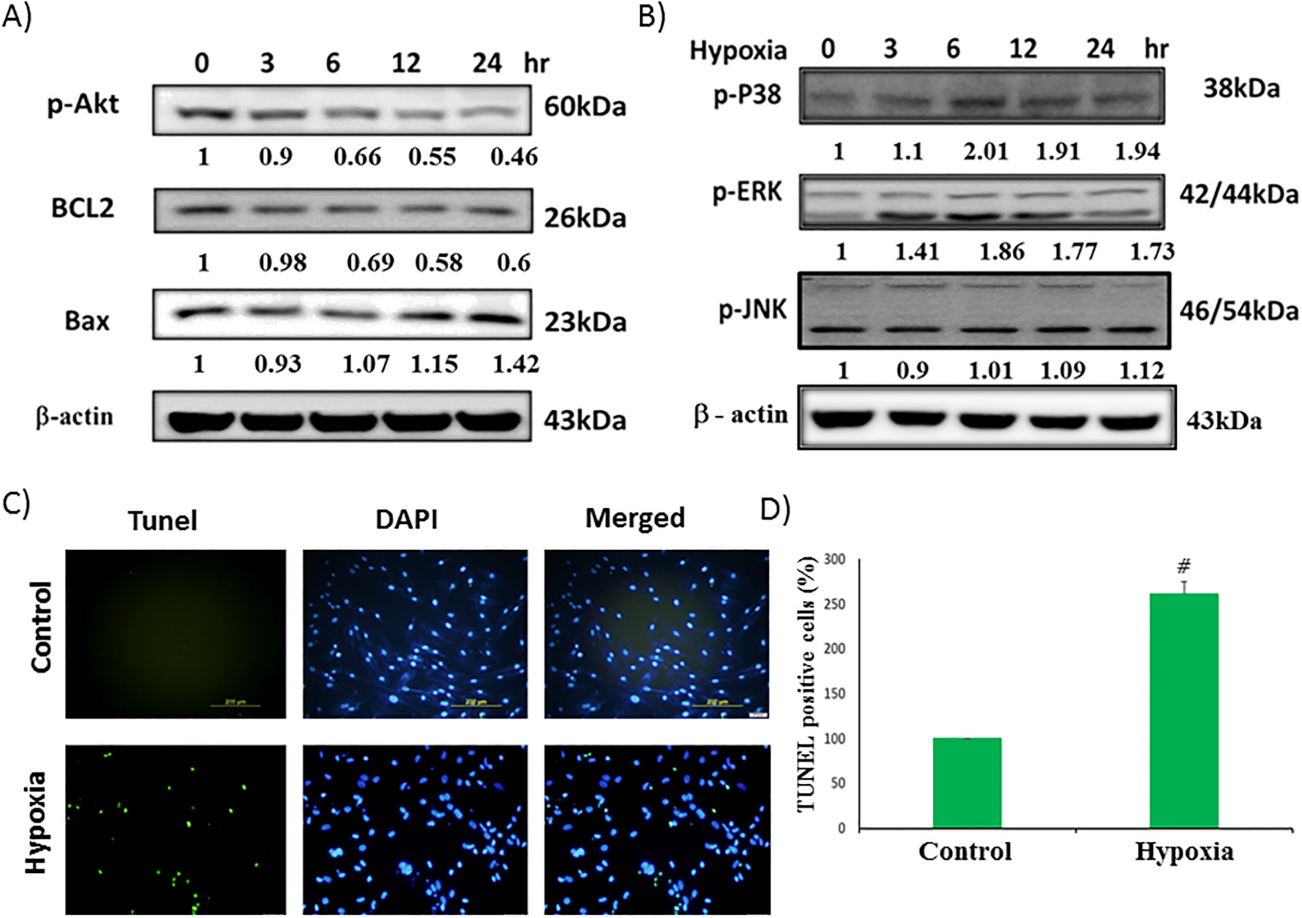
Effect of hypoxia-induced apoptosis and activation of MAPK family proteins, and survival proteins in H9c2 cells. (A) Survival proteins for 0-24h. (B) MAPK family (p-ERK, p-JNK, p-P38). (C)Fluorescence images show the cells stained with 4, 6-diamidino-2-phenylindole (DAPI) and stained using terminal deoxynucleotidyl transferase dUTP-mediated nick-end labeling (TUNEL) assay. (D) Graphical representation of TUNEL assay. Data showed the means ± SEM of 3 independent analyses. *P<0.05 vs. hypoxia treatment.

We examined the survival protein (p-Akt^ser473^), Bcl2, and pro-apoptotic protein (Bax) in different time of hypoxia condition. The results showed that hypoxia downregulated the survival protein (Fig 2A) whereas pro-apoptotic protein was upregulated. To analyze hypoxia induced cell apoptosis in cardiac myocyte, TUNEL assay was performed. After 24h incubation in hypoxic chamber, we observed a significant increase in apoptotic bodies (Fig 2C).

### Roles of MAPK family proteins in hypoxia induced H9c2 cell apoptosis

To investigate whether MAPK family proteins are involved in the hypoxia-induced H9c2 apoptosis, we examined the expression levels of MAPK family proteins by Western blot. Our results showed that the expression of p-ERK, and p-P38 were increased after treatment with hypoxia for 0-24h, whereas pJNK expression was not increased notably (Fig 2B).

### Effects of HDL on hypoxia-induced NADPH oxidase complex and antioxidant enzyme

**Fig 3:**
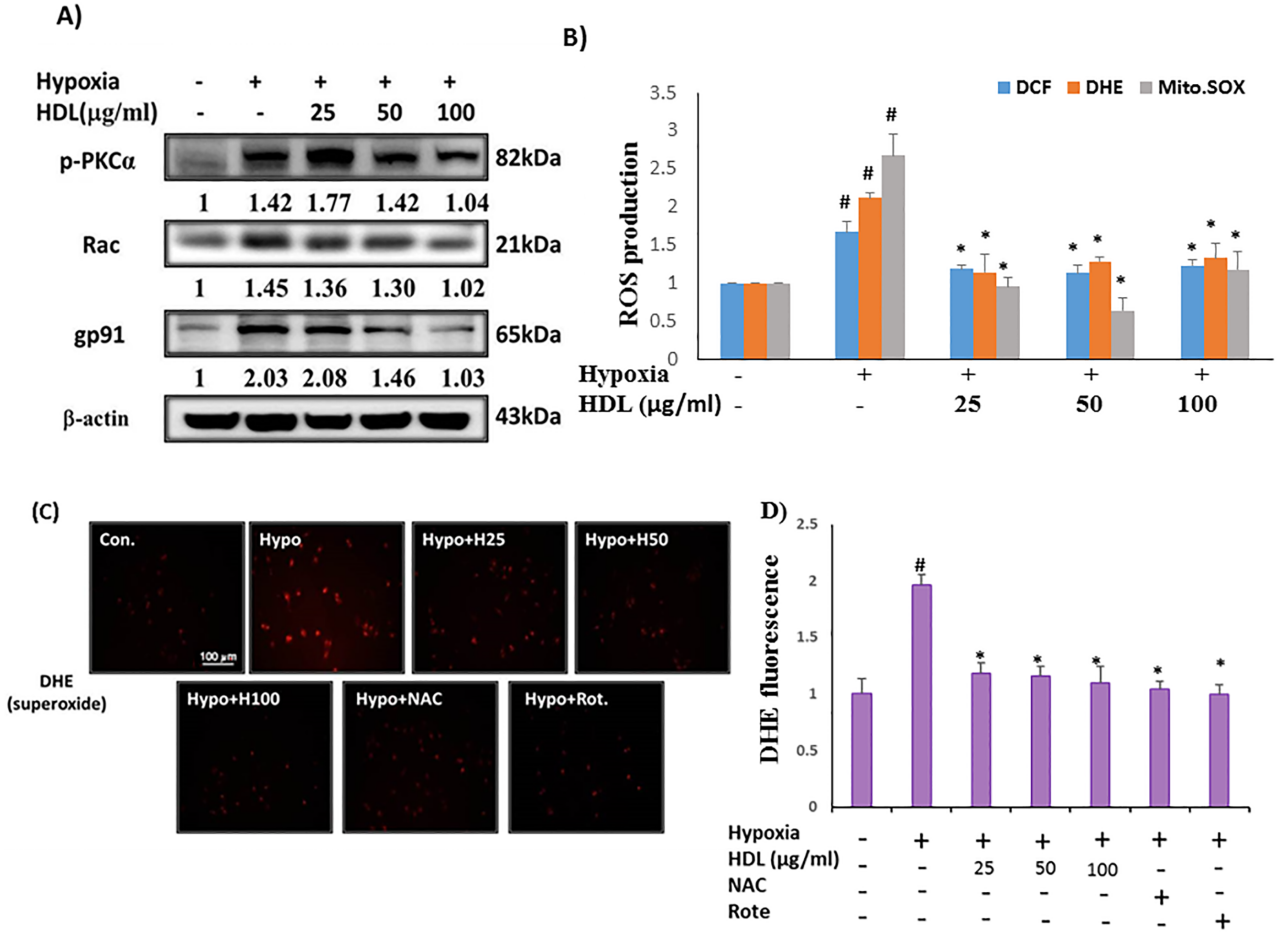
Inhibitory effect of HDL on hypoxia induced ROS production in H9c2 cells. (A) Representative western blots. ‘Image J’ software was used to calculate the expression level of protein (B). ROS was examined by DCF-AM (10μM), DHE (5μM), and MitoSOX^™^ (5μM). Fluorescence intensity of cells was measured by flow cytometry. (C) Neonatal cardiomyocytes were treated with HDL (25–100μg/ml) for 2h, or NAC (500 μM), roteone (5 μM), and then incubated with 1%hypoxia for an additional 24h, and followed by 1h incubation with DHE (10μM). Fluorescence intensity of cells was measured by immunofluorescence microscopy (Olympus CKK53). Data showed the means ± SEM of 3 independent analyses.^#^P<0.05 comparison of control and hypoxia groups, *P<0.05 HDL/ NAC treated groups vs. hypoxia treatment.

The effects of HDL (25, 50 and100μg/ml) on expression levels of p-PKC- α, gp91phox and Rac-1 were examined by Western blotting in H9c2 cells. We found that hypoxia treatment of H9c2 cells resulted in the increase of NADPH oxidase activity. However, pretreatment with HDL (25–100μg/ml) led to a dose-dependent reduction in gp91phox and Rac-1 protein expression (Fig 3A). As shown in Fig 3A, incubation of H9c2 cells with hypoxia resulted in significant phosphorylation of protein kinase C (p-PKC-α) and HDL attenuated the expression of p-PKC-α. We further investigated the effects of HDL on generation of ROS, a potential factor related to hypoxia-induced H9c2 cells injury, using hydroxyl radical sensitive probe 2’,7’-dichlorofluorescein acetoxymethyl ester (DCF-AM), superoxide sensitive probe dihydroethidium (DHE) and MitoSOX^™^ Red mitochondrial superoxide indicator. The production of ROS and superoxide generations were examined by flow cytometry. Results revealed a significant reduction of ROS in H9c2 cells pretreated with HDL (25–100μg/ml) in a dose-dependent manner (Fig 3B).

### Sustain exposure of HDL can reduce NADPH oxidase activity in neonatal cardiomyocytes treated with hypoxia

To explore the effects of HDL on NADPH oxidase, a major source of ROS in H9c2 cells, we measured the formation of superoxide by using superoxide sensitive probe dihydroethidium (DHE). As shown in Fig 3C, neonatal cardiomyocytes pretreated with HDL (25–100μg/ml) for 2h before exposure to hypoxia for 24h, we used superoxide sensitive probe dihydroethidium (DHE) to confirm by microscopic observation. The results showed that hypoxia induced superoxide generation was reduced in HDL (25–100μg/ml) pretreated cells, similar results were observed by the treatments with anti-oxidant (NAC, 500 μM) and mitochondrial superoxide inhibitor (Rotenone, 5 μM).

### Effect of HDL on hypoxia-induced MAPK protein phosphorylation

**Fig 4:**
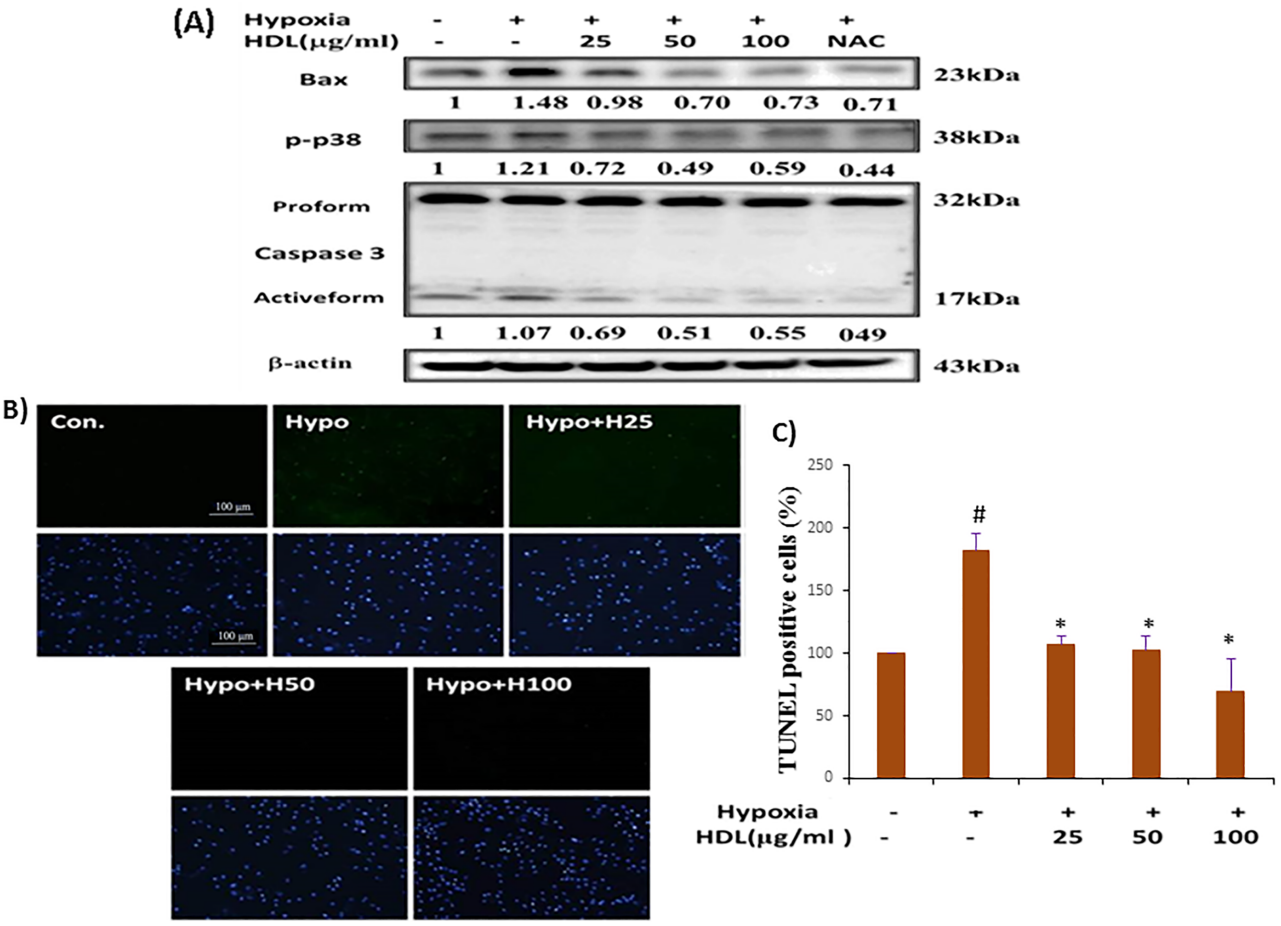
Inhibitory effect of HDL on hypoxia induced MAPK and apoptotic protein expression in H9c2 cells. (A). Representative western blots. ‘Image J’ software was used to calculate the expression level of protein. (B) Fluorescence images show the cells stained with 4,6-diamidino-2-phenylindole (DAPI) (upper panel) and stained using terminal deoxynucleotidyl transferase dUTP-mediated nick-end labeling (TUNEL) assay (bottom panel), and photomicrographs were from immunofluorescence microscopy (Olympus CKK53). C). Graphical representation of TUNEL assay. ^#^P<0.05 comparison of control and hypoxia groups, *P<0.05 HDL treated groups vs. hypoxia treatment.

Incubation of H9c2 cells with hypoxia resulted in significant phosphorylation of p38, and ERK, but less change in the expression of pJNK. Therefore, we examined whether HDL could inhibit MAPK proteins (p-p38 and pERK) activation. H9c2 cells were treated with HDL (25–100μg/ml) for 2h before exposure to hypoxia for 24h, our data showed HDL can downregulate the phosphorylation of p38 only (Fig 4A). Whereas significant reduction was not observed in the expression of pERK (data not shown). Thus, HDL pretreatment could suppress hypoxia mediated apoptosis by downregulating p38 MAPK.

### HDL attenuated the apoptotic effects of hypoxia by regulating Bcl2 family protein, and the activation of casepase3

Immunoblotting studies demonstrated that hypoxia downregulated the anti-apoptotic and survival proteins (Bcl2, p-Akt^ser473^), also upregulated the pro-apoptotic protein (Bax), whereas HDL pretreatment effectively reverted these effects. Since activated caspase 3 is a key factor in the execution of mitochondrial apoptosis [20], we subsequently determined the expression of caspase 3, both pro-form and active-form and Bax by immunoblotting (Fig 4A). The data showed that active caspase 3 was significantly increased in cells that had been treated with hypoxia whereas suppressed in cells pretreated with HDL. Furthermore, at cellular level, the anti-apoptotic effects of HDL on hypoxia-induced cell death was evaluated by TUNEL and DAPI staining assays. As shown in Fig 4B & 4C, hypoxia treated cells showed a typical features of apoptosis, including the formation of condensed nuclei. However, apoptosis was not observed in the HDL-pretreated H9c2 cells. As described above, both results of cell viability assay and phenotypic observation of apoptosis under microscopy suggested that HDL is a potent inhibitor of hypoxia-induced apoptosis in cultured H9c2 cells. NAC (500μM) could suppress TUNEL-positive cell, induced by hypoxia.

## Discussion

Hypoxic condition is one of the common causes of cell damage, which is implicated in many pathologic conditions which include stroke, myocardial infarction (MI), diabetes mellitus and multiple organ failure [21, 22]. Plaques formation in the heart's arteries is known to lead to myocardial remodeling and myocardial hypoxia [23]. Angiotensin II (Ang II) was reported to mediate hypoxia-induced caspase-3 activation through ERK pathway in primary cortical neuronal cultures [4]. However, in the present study, we found that hypoxia induced apoptosis was successfully reverted by HDL pretreatment in H9c2 cells. In human umbilical vein endothelial cells, the mitochondrial pathway of apoptosis was shown to be blocked by HDL [24]. Tumor necrosis factor-α-induced apoptosis was reported to be inhibited by HDL in endothelial cells [25].

Our study was extended to explore the mechanisms of hypoxia-induced cell apoptosis in heart, focusing on the NADPH oxidase-generated ROS induced signaling and also concentrated on the therapeutic potential of inhibiting NADPH oxidase in hypoxia-exposed cardiac cells. The effects of ang II are mediated by two distinct receptors, referred to as the Ang II type-1 (AT1) and type-2 (AT2) receptor subtypes. AT1 receptor is dependent on the cell and organ type, binding of Ang II with AT1 receptor leads to cellular contraction, hypertrophy, proliferation, and/or apoptosis and importantly, the generation and release of ROS [26]. Upon activation of AT1 receptor, p47phox becomes phosphorylated and translocated to the membrane, where p47phox forms a complex with gp91phox and p22phox and the assembly performs as an active oxidase [27]. The effect of hypoxia resulted in increased expression of AT1 and NADPH oxidase proteins (Fig 1). Various protein kinases have been identified to be involved in the regulation of NADPH oxidase activity [28, 29], among them the protein kinase C (PKC) family was found to play a critical role [30–32]. Our results proved that phosphorylation of PKC α and δ was increased under hypoxia condition. Furthermore, hypoxia activated NADPH oxidase via AT1 receptor was also evidenced. SOD is known to protect cells against superoxide-mediated cytotoxicity by rapidly dismutating O2– to H2O2. Treatment of hypoxia decreased Cu/Zn-SOD and Mn-SOD protein expression level (Fig 1). Whereas HDL pretreated cells resulted in decreased ROS generation, and subsequently attenuated hypoxia-impaired superoxide dismutase (SOD) activity and suppressed ROS-induced intracellular signaling pathways (Fig 3). HDLs from healthy persons have been found to activate endothelial nitric oxide (NO) generation which decreases the production of endothelial ROS [33]. Many studies have reported that MAPK signaling pathway is stimulated by hypoxia. [34–38]. Here we showed that hypoxia increased the expression of p-P38 and p-ERK, but not p-JNK significantly and HDL pretreatment could protect these effects caused by hypoxia only by downregulating p-P38 MAPK.

## Conclusion

**Fig 5:**
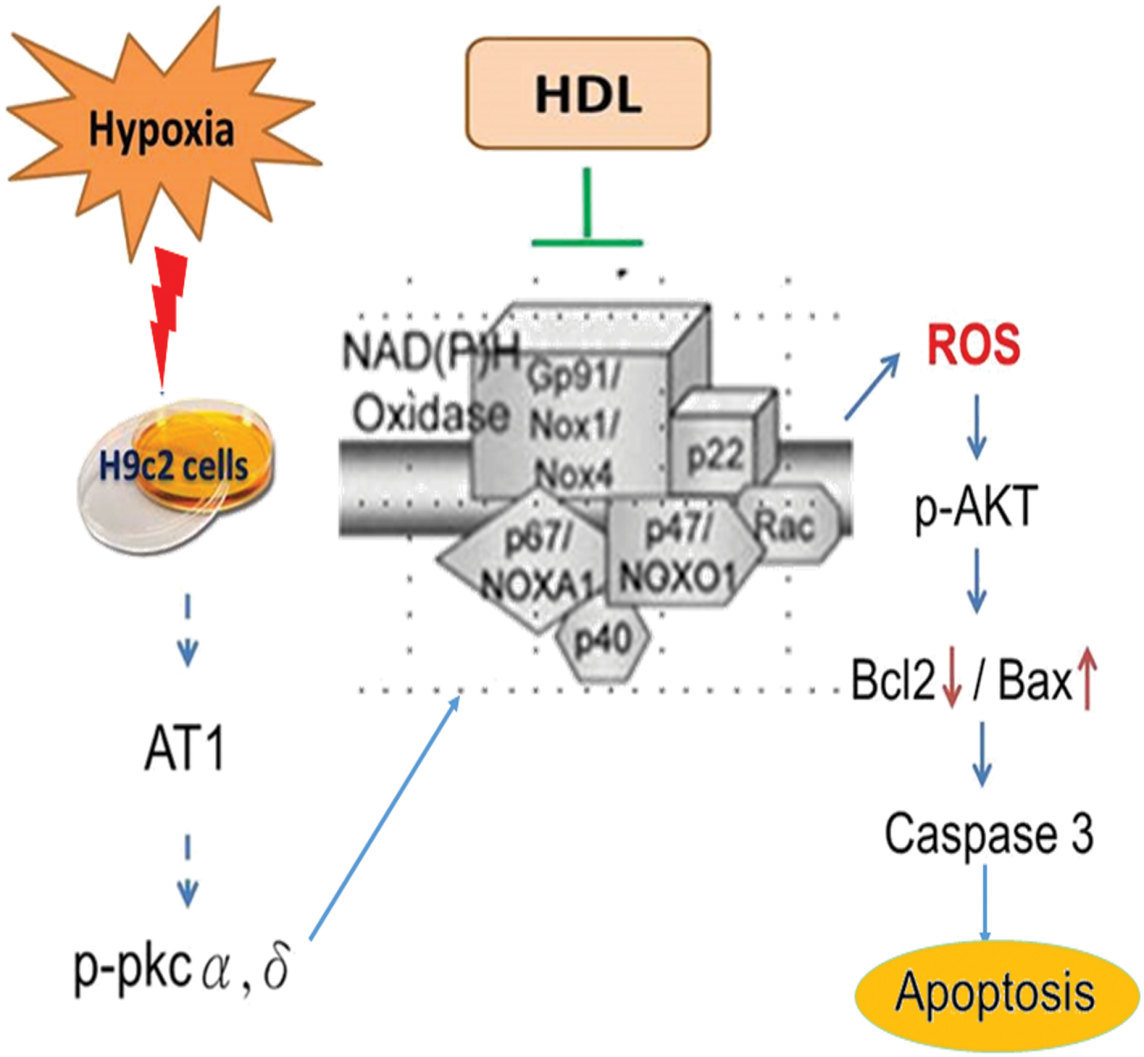
Schematic representation of the effect of hypoxia and the preventive role of HDL on H9c2 cells. Hypoxia induces ROS mediated apoptosis through the overexpression of AT1 and PKC, which is prevented by the treatment of HDL.

Our *in vitro* studies revealed that HDL could prevent from hypoxia-induced cardiomyocyte apoptosis and oxidative dysfunction via modulating mitochondria dependent pathway (Fig 5). Further, the findings provided an insight of underlying molecular mechanism, which leads to the progression of cardiovascular disease. In H9c2 cells, it was confirmed that hypoxia triggered AT1 receptor and NADPH oxidase activity. Future direction of the present study would be whether the protective effect of HDL against hypoxia-induced apoptosis is mediated by the downregulation of AT1receptor or the involvement of any other receptors and also the specific component of HDL such as Apo A-1, SR-B1 is responsible for the activity of HDL. The findings would pave the way to the discovery of accurate drugs for cardiovascular diseases.

## References

[1] Chroni A, Leondaritis G, Karlsson H. Lipids and lipoproteins in atherosclerosis. Journal of lipids. 2011;2011:160104 doi: 10.1155/2011/160104 . 22523690 10.1155/2011/160104 PMC3317094

[2] Li X, Fang P, Li Y, Kuo YM, Andrews AJ, Nanayakkara G, et al Mitochondrial Reactive Oxygen Species Mediate Lysophosphatidylcholine-Induced Endothelial Cell Activation. Arteriosclerosis, thrombosis, and vascular biology. 2016;36(6):1090–100. doi: 10.1161/ATVBAHA.115.306964 . 27127201 10.1161/ATVBAHA.115.306964 PMC4882253

[3] Giordano FJ. Oxygen, oxidative stress, hypoxia, and heart failure. J Clin Invest. 2005;115(3):500–8. Epub 2005/03/15. doi: 10.1172/JCI200524408 . 15765131 10.1172/JCI200524408 PMC1052012

[4] Chiu CZ, Wang BW, Chung TH, Shyu KG. Angiotensin II and the ERK pathway mediate the induction of myocardin by hypoxia in cultured rat neonatal cardiomyocytes. Clin Sci (Lond). 2010;119(7):273–82. Epub 2010/05/08. doi: 10.1042/CS20100084 . 20446923 10.1042/CS20100084 PMC2890999

[5] Huang Y, Hickey RP, Yeh JL, Liu D, Dadak A, Young LH, et al Cardiac myocyte-specific HIF-1alpha deletion alters vascularization, energy availability, calcium flux, and contractility in the normoxic heart. FASEB J. 2004;18(10):1138–40. Epub 2004/05/11. doi: 10.1096/fj.04-1510fje 15132980 10.1096/fj.04-1510fje

[6] Giordano FJ, Johnson RS. Angiogenesis: the role of the microenvironment in flipping the switch. Curr Opin Genet Dev. 2001;11(1):35–40. Epub 2001/02/13. . 11163148 10.1016/s0959-437x(00)00153-2

[7] Ryan HE, Lo J, Johnson RS. HIF-1 alpha is required for solid tumor formation and embryonic vascularization. EMBO J. 1998;17(11):3005–15. Epub 1998/06/26. doi: 10.1093/emboj/17.11.3005 . 9606183 10.1093/emboj/17.11.3005 PMC1170640

[8] Carmeliet P, Dor Y, Herbert JM, Fukumura D, Brusselmans K, Dewerchin M, et al Role of HIF-1alpha in hypoxia-mediated apoptosis, cell proliferation and tumour angiogenesis. Nature. 1998;394(6692):485–90. Epub 1998/08/11. doi: 10.1038/28867 . 9697772 10.1038/28867

[9] Iyer NV, Kotch LE, Agani F, Leung SW, Laughner E, Wenger RH, et al Cellular and developmental control of O2 homeostasis by hypoxia-inducible factor 1 alpha. Genes Dev. 1998;12(2):149–62. Epub 1998/03/07. . 9436976 10.1101/gad.12.2.149 PMC316445

[10] Fearon IM, Faux SP. Oxidative stress and cardiovascular disease: novel tools give (free) radical insight. J Mol Cell Cardiol. 2009;47(3):372–81. Epub 2009/06/02. . 19481547 10.1016/j.yjmcc.2009.05.013

[11] Davies KJ. Oxidative stress: the paradox of aerobic life. Biochem Soc Symp. 1995;61:1–31. Epub 1995/01/01. . 8660387 10.1042/bss0610001

[12] Ide T, Tsutsui H, Kinugawa S, Utsumi H, Kang D, Hattori N, et al Mitochondrial electron transport complex I is a potential source of oxygen free radicals in the failing myocardium. Circ Res. 1999;85(4):357–63. Epub 1999/08/24. . 10455064 10.1161/01.res.85.4.357

[13] Miano JM. Serum response factor: toggling between disparate programs of gene expression. J Mol Cell Cardiol. 2003;35(6):577–93. Epub 2003/06/06. . 12788374 10.1016/s0022-2828(03)00110-x

[14] Wang D, Chang PS, Wang Z, Sutherland L, Richardson JA, Small E, et al Activation of cardiac gene expression by myocardin, a transcriptional cofactor for serum response factor. Cell. 2001;105(7):851–62. Epub 2001/07/06. . 11439182 10.1016/s0092-8674(01)00404-4

[15] Zhang X, Azhar G, Zhong Y, Wei JY. Identification of a novel serum response factor cofactor in cardiac gene regulation. J Biol Chem. 2004;279(53):55626–32. Epub 2004/10/20. doi: 10.1074/jbc.M405945200 . 15492011 10.1074/jbc.M405945200

[16] Wang DZ, Li S, Hockemeyer D, Sutherland L, Wang Z, Schratt G, et al Potentiation of serum response factor activity by a family of myocardin-related transcription factors. Proc Natl Acad Sci U S A. 2002;99(23):14855–60. Epub 2002/10/25. doi: 10.1073/pnas.222561499 12397177 10.1073/pnas.222561499 PMC137508

[17] Navab M, Reddy ST, Van Lenten BJ, Fogelman AM. HDL and cardiovascular disease: atherogenic and atheroprotective mechanisms. Nature reviews Cardiology. 2011;8(4):222–32. doi: 10.1038/nrcardio.2010.222 21304474 10.1038/nrcardio.2010.222

[18] Lin CJ, Lai CK, Kao MC, Wu LT, Lo UG, Lin LC, et al Impact of cholesterol on disease progression. BioMedicine. 2015;5(2):7 doi: 10.7603/s40681-015-0007-8 26048694 10.7603/s40681-015-0007-8 PMC4502043

[19] Vaisar T, Mayer P, Nilsson E, Zhao XQ, Knopp R, Prazen BJ. HDL in humans with cardiovascular disease exhibits a proteomic signature. Clin Chim Acta. 2010;411(13–14):972–9. Epub 2010/03/24. doi: 10.1016/j.cca.2010.03.023 . 20307520 10.1016/j.cca.2010.03.023 PMC2862883

[20] Narula J, Pandey P, Arbustini E, Haider N, Narula N, Kolodgie FD, et al Apoptosis in heart failure: release of cytochrome c from mitochondria and activation of caspase-3 in human cardiomyopathy. Proceedings of the National Academy of Sciences of the United States of America. 1999;96(14):8144–9. Epub 1999/07/08. . 10393962 10.1073/pnas.96.14.8144 PMC22202

[21] Halterman MW, Federoff HJ. HIF-1alpha and p53 promote hypoxia-induced delayed neuronal death in models of CNS ischemia. Exp Neurol. 1999;159(1):65–72. Epub 1999/09/16. doi: 10.1006/exnr.1999.7160 10486175 10.1006/exnr.1999.7160

[22] Kim JY, Ahn HJ, Ryu JH, Suk K, Park JH. BH3-only protein Noxa is a mediator of hypoxic cell death induced by hypoxia-inducible factor 1alpha. J Exp Med. 2004;199(1):113–24. Epub 2003/12/31. doi: 10.1084/jem.20030613 14699081 10.1084/jem.20030613 PMC1887730

[23] Lee SH, Wolf PL, Escudero R, Deutsch R, Jamieson SW, Thistlethwaite PA. Early expression of angiogenesis factors in acute myocardial ischemia and infarction. N Engl J Med. 2000;342(9):626–33. Epub 2000/03/04. doi: 10.1056/NEJM200003023420904 . 10699162 10.1056/NEJM200003023420904

[24] Nofer JR, Levkau B, Wolinska I, Junker R, Fobker M, von Eckardstein A, et al Suppression of endothelial cell apoptosis by high density lipoproteins (HDL) and HDL-associated lysosphingolipids. The Journal of biological chemistry. 2001;276(37):34480–5. doi: 10.1074/jbc.M103782200 . 11432865 10.1074/jbc.M103782200

[25] Sugano M, Tsuchida K, Makino N. High-density lipoproteins protect endothelial cells from tumor necrosis factor-alpha-induced apoptosis. Biochemical and biophysical research communications. 2000;272(3):872–6. doi: 10.1006/bbrc.2000.2877 . 10860844 10.1006/bbrc.2000.2877

[26] Nickenig G, Harrison DG. The AT(1)-type angiotensin receptor in oxidative stress and atherogenesis: part I: oxidative stress and atherogenesis. Circulation. 2002;105(3):393–6. Epub 2002/01/24. . 11804998 10.1161/hc0302.102618

[27] Zeng Q, Han Y, Bao Y, Li W, Li X, Shen X, et al 20-HETE increases NADPH oxidase-derived ROS production and stimulates the L-type Ca2+ channel via a PKC-dependent mechanism in cardiomyocytes. American journal of physiology Heart and circulatory physiology. 2010;299(4):H1109–17. Epub 2010/08/03. doi: 10.1152/ajpheart.00067.2010 . 20675568 10.1152/ajpheart.00067.2010 PMC2957347

[28] El Benna J, Faust RP, Johnson JL, Babior BM. Phosphorylation of the respiratory burst oxidase subunit p47phox as determined by two-dimensional phosphopeptide mapping. Phosphorylation by protein kinase C, protein kinase A, and a mitogen-activated protein kinase. J Biol Chem. 1996;271(11):6374–8. Epub 1996/03/15. . 8626435 10.1074/jbc.271.11.6374

[29] McPhail LC, Qualliotine-Mann D, Waite KA. Cell-free activation of neutrophil NADPH oxidase by a phosphatidic acid-regulated protein kinase. Proc Natl Acad Sci U S A. 1995;92(17):7931–5. Epub 1995/08/15. . 7644515 10.1073/pnas.92.17.7931 PMC41260

[30] Wolfson M, McPhail LC, Nasrallah VN, Snyderman R. Phorbol myristate acetate mediates redistribution of protein kinase C in human neutrophils: potential role in the activation of the respiratory burst enzyme. J Immunol. 1985;135(3):2057–62. Epub 1985/09/01. . 3160785

[31] Combadiere C, el Benna J, Pedruzzi E, Hakim J, Perianin A. Stimulation of the human neutrophil respiratory burst by formyl peptides is primed by a protein kinase inhibitor, staurosporine. Blood. 1993;82(9):2890–8. Epub 1993/11/01. . 8219237

[32] Nauseef WM, Volpp BD, McCormick S, Leidal KG, Clark RA. Assembly of the neutrophil respiratory burst oxidase. Protein kinase C promotes cytoskeletal and membrane association of cytosolic oxidase components. J Biol Chem. 1991;266(9):5911–7. Epub 1991/03/25. . 1848559

[33] Zewinger S, Drechsler C, Kleber ME, Dressel A, Riffel J, Triem S, et al Serum amyloid A: high-density lipoproteins interaction and cardiovascular risk. European heart journal. 2015;36(43):3007–16. doi: 10.1093/eurheartj/ehv352 26248570 10.1093/eurheartj/ehv352

[34] Bogoyevitch MA, Gillespie-Brown J, Ketterman AJ, Fuller SJ, Ben-Levy R, Ashworth A, et al Stimulation of the stress-activated mitogen-activated protein kinase subfamilies in perfused heart. p38/RK mitogen-activated protein kinases and c-Jun N-terminal kinases are activated by ischemia/reperfusion. Circ Res. 1996;79(2):162–73. Epub 1996/08/01. . 8755992 10.1161/01.res.79.2.162

[35] Cook SA, Sugden PH, Clerk A. Activation of c-Jun N-terminal kinases and p38-mitogen-activated protein kinases in human heart failure secondary to ischaemic heart disease. J Mol Cell Cardiol. 1999;31(8):1429–34. Epub 1999/07/29. doi: 10.1006/jmcc.1999.0979 10423341 10.1006/jmcc.1999.0979

[36] Sugden PH, Clerk A. "Stress-responsive" mitogen-activated protein kinases (c-Jun N-terminal kinases and p38 mitogen-activated protein kinases) in the myocardium. Circ Res. 1998;83(4):345–52. Epub 1998/08/29. . 9721691 10.1161/01.res.83.4.345

[37] Nakano A, Baines CP, Kim SO, Pelech SL, Downey JM, Cohen MV, et al Ischemic preconditioning activates MAPKAPK2 in the isolated rabbit heart: evidence for involvement of p38 MAPK. Circ Res. 2000;86(2):144–51. Epub 2000/02/10. . 10666409 10.1161/01.res.86.2.144

[38] Hsieh SR, Cheng WC, Su YM, Chiu CH, Liou YM. Molecular targets for anti-oxidative protection of green tea polyphenols against myocardial ischemic injury. BioMedicine. 2014;4:23 doi: 10.7603/s40681-014-0023-0 . 25520936 10.7603/s40681-014-0023-0 PMC4264984

